# Vitamin D Status in Patients at the Department of Internal, Autoimmune, and Metabolic Diseases—A Descriptive Cross-Sectional Study

**DOI:** 10.3390/biomedicines13092170

**Published:** 2025-09-05

**Authors:** Zuzanna Pawlus, Patryk Mosiołek, Karolina Bierć, Aleksandra Pilśniak, Agata Janoska-Gawrońska, Michał Holecki

**Affiliations:** 1 Student Scientific Society at the Department of Internal, Autoimmune, and Metabolic Diseases, Faculty of Medical Sciences, Medical University of Silesia, 40-055 Katowice, Poland; zuzpaw1999@gmail.com (Z.P.); patrykjaroslaw@gmail.com (P.M.); bierckarolina@gmail.com (K.B.); 2 Department of Internal Medicine, Autoimmune and Metabolic Diseases, Faculty of Medical Sciences, Medical University of Silesia, 40-055 Katowice, Poland; agata.janoska@op.pl

**Keywords:** vitamin D, hypovitaminosis D, length of hospital stay, supplementation

## Abstract

**Background:** Vitamin D plays a crucial role in numerous physiological systems, including the muscular, immune, and endocrine systems, as well as calcium and bone homeostasis. Vitamin D deficiency, a global health problem affecting billions of people, is associated with adverse health outcomes and can serve as a marker for health status, disease severity, and length of hospital stay (LOS). Nutritional supplementation has been shown to be highly beneficial and is strongly recommended. **Methods:** In this study, the medical records of 834 hospitalized patients were retrospectively analyzed, with a focus on 25-hydroxyvitamin D [25(OH)D] levels. The patients were divided into groups based on serum vitamin D levels: deficient, suboptimal, adequate, and high. Correlations between vitamin D levels, LOS, laboratory parameters, comorbidities, seasonal variations, medical diagnosis of deficiency, and supplementation were examined. **Results:** Vitamin D deficiency was common and associated with a longer LOS. Women had higher vitamin D levels than men. No seasonal variation was observed. Only 14% of the patients took supplements before hospitalization, and 61% of the patients with vitamin D deficiency received supplementation. **Conclusions:** Routine vitamin D screening, tailored supplementation, and increased public awareness are essential to reduce hospitalizations, improve treatment outcomes, and promote independence among at-risk groups.

## 1. Introduction

Vitamin D plays a crucial role in the human body for the muscular, immune, endocrine, and central nervous systems as well as for calcium and bone homeostasis [1].

Vitamin D deficiency is a global health problem affecting billions of people worldwide and is associated with a variety of negative health effects, both skeletal and otherwise [2,3]. Epidemiological studies have shown significant associations between low vitamin D levels and chronic diseases such as cardiovascular disease, autoimmune disease, cancer, type 2 diabetes (T2DM), and Alzheimer’s disease [1,4,5].

The effects of vitamin D deficiency, characterized by muscle weakness, bone pain, and fragility fractures, are well known [6]. However, increasing attention is being paid to non-skeletal roles. Vitamin D plays a role in regulating the immune system, cardiovascular homeostasis, and metabolism [1,4]. There is evidence that maintaining adequate vitamin D levels may reduce the risk of arterial hypertension (AH), myocardial infarction, atrial fibrillation, and the progression from prediabetes to diabetes [5].

Vitamin D deficiency is particularly common in older adults, with hypovitaminosis D affecting 50–90% of this population. This is due to age-related factors such as impaired skin synthesis, lower dietary intake, and reduced hydroxylation in the liver and kidneys [3,4]. Low serum vitamin D levels in hospitalized older adults are associated with higher comorbidity and longer hospital stays, suggesting that hypovitaminosis D may serve as a marker of overall health status and disease severity [1].

Vitamin D is mainly synthesized in the skin by exposure to ultraviolet light [5]; other sources are food and dietary supplements [6]. However, the efficiency of skin synthesis depends on factors such as skin pigmentation, age, latitude, and season and time of day, making it difficult to estimate the amount of sunlight required to meet vitamin D needs [7]. With increasing age, the metabolism of vitamin D and calcium is impaired. The prevalence of poor renal function and malabsorption syndromes in the elderly is increased, resulting in abnormal levels of 1,25-dihydroxyvitamin D [1].

Geographic and seasonal variations also play an important role in vitamin D status. Populations living at higher latitudes or experiencing long winters often have lower serum vitamin D levels, especially in regions where food fortification is limited [8]. The prevalence of vitamin D deficiency has increased dramatically worldwide over the past decade, with the condition increasingly affecting both children and adults [9].

The standard method for assessing vitamin D status is to measure serum concentrations of 25-hydroxyvitamin D (25(OH)D), the major circulating form of the vitamin [10,11]. This biomarker provides a reliable indicator of vitamin D status and allows physicians to identify at-risk individuals and recommend appropriate interventions. For populations with a high prevalence of deficiency, such as the elderly or inhabitants of geographic regions with insufficient ultraviolet B (UVB) exposure, routine serum 25(OH)D testing is strongly recommended [12].

Vitamin D supplementation has shown promising benefits in reducing blood pressure, managing hyperglycemia, and potentially reducing the risk of cardiovascular disease and cancer [1,13]. It also plays a role in improving overall musculoskeletal health, reducing the risk of falls, and alleviating symptoms of depression [1].

The aim of this study was to examine the levels of vitamin D (25(OH)D) among patients hospitalized in 2022 at the Department of Internal, Autoimmune, and Metabolic Diseases. We examined the correlation between vitamin D levels and the length of hospitalization, other laboratory parameters measured at admission, the most common diseases, the season of hospitalization, as well as physician diagnosis of deficiency and supplementation practices.

## 2. Materials and Methods

### 2.1. Study Population

In 2022, a total of 996 patients were admitted to the Department of Internal, Autoimmune, and Metabolic Diseases in Katowice, Poland. A total of 834 patients were included in the study. A total of 162 patients were excluded due to missing data on vitamin D status.

### 2.2. Methods

We retrospectively examined the electronic medical records of 834 patients admitted to the Department of Internal, Autoimmune, and Metabolic Diseases in 2022. Data collected included patient demographics (age, sex, body weight, height, body mass index (BMI)) as well as length of hospitalization (LOS), month of hospitalization, medical diagnoses (T2DM, AH, osteoporosis), laboratory results measured on admission, and diagnosis of vitamin D deficiency and recommendation for vitamin D supplementation. Vitamin D concentration was measured in the hospital laboratory using an enzyme-linked immunosorbent assay (ELISA). Patients were divided into 4 groups according to the diagnostic threshold for serum 25(OH)D concentration approved for Central Europe: deficient, suboptimal, optimal, and high serum vitamin D levels [12].

### 2.3. Statistical Analysis

The data were analyzed using the statistical software STATISTICA 13.3.721.1. The Shapiro–Wilk test was used to assess the normality of the distribution of the analyzed variables. Normally distributed data were compared using Student’s *t*-test or analysis of variance (ANOVA). Non-parametric data were compared using the Mann–Whitney U-test or the Kruskal–Wallis test. The variables analyzed were expressed as mean ± SD (for normally distributed data) and as median, lower quartile, and upper quartile (for non-parametric data). A probability value (*p*-value) ≤ 0.05 was considered significant, followed by univariate and multiple linear regression analysis examining the correlation between length of hospitalization and serum 25(OH)D level and BMI and serum 25(OH)D level.

### 2.4. Bioethics Committee

The data were fully anonymized. In accordance with the regulations of the Medical University of Silesia in Katowice, our study is a retrospective study, which does not constitute a medical experiment and for which no evaluation by the Bioethics Committee and no patient consent were required.

## 3. Results

**Figure 1:**
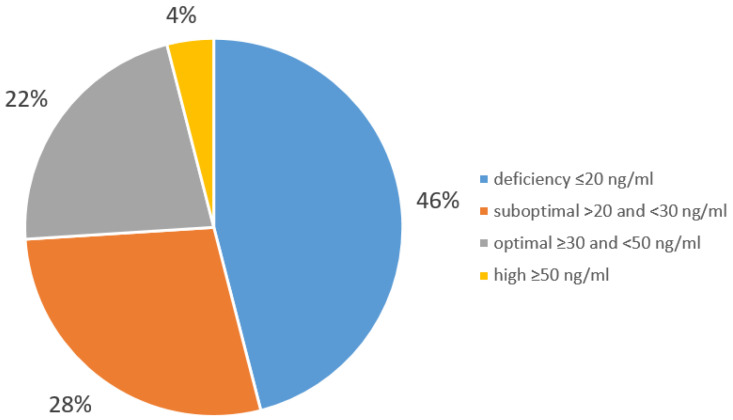
Vitamin D status of patients from the Department of Internal, Autoimmune, and Metabolic Diseases (*n* = 834).

In this study, the vitamin D concentrations were measured in all patients, who were divided into 4 groups based on the diagnostic threshold for serum 25(OH)D concentration established for Central Europe. [12] The first group (deficiency group) consisted of patients with vitamin D deficiency (N = 382), defined as a serum 25(OH)D concentration of 20 ng/mL or less. The second group (suboptimal group) consisted of patients with a suboptimal vitamin D status (N = 236), defined as a serum 25(OH)D concentration between >20 and <30 ng/mL. Patients with adequate vitamin D status (N = 186), defined as serum 25(OH)D concentration ≥30 and <50 ng/mL, were assigned to the third group, and patients with high vitamin D levels (N = 30)—serum 25(OH)D defined as equal to or above 50 ng/mL—were included in the fourth group. The proportion of patients across the different vitamin D level categories is illustrated in Figure 1.

**Figure 2:**
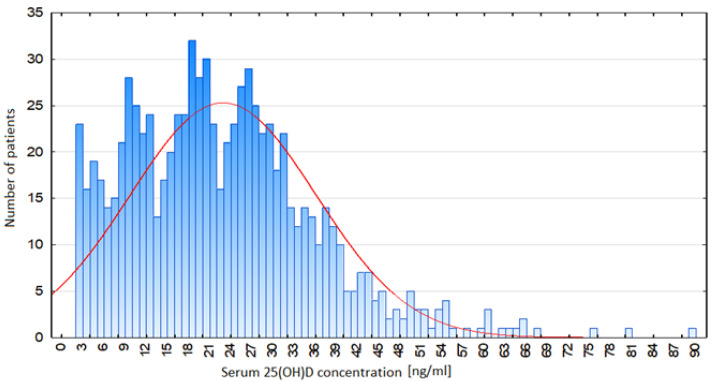
Distribution of serum 25(OH)D concentration among patients.

The distribution of 25(OH)D concentration in the serum of the patients is presented in Figure 2.

The mean vitamin D concentration was 22.76 ng/mL, the median was 21.3 ng/mL, the minimum value was less than 3 ng/mL, and the maximum value was 89.1 ng/mL. Women had higher vitamin D concentrations (mean: 24.4 ng/mL). The baseline clinical and laboratory data of the study group are presented in Table 1.

**Figure 3:**
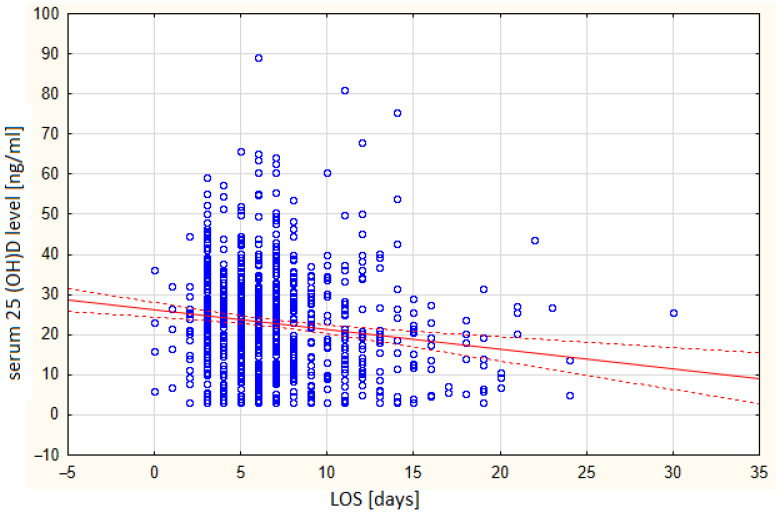
Correlation between LOS and serum vitamin D levels. Confidence interval—95%. r = 0.1453.

The average age of the patients was 63 years, with no significant differences between the groups (*p* = 0.78). A significant statistical correlation was found between the length of hospitalization and vitamin D levels (Figure 3).

**Figure 4:**
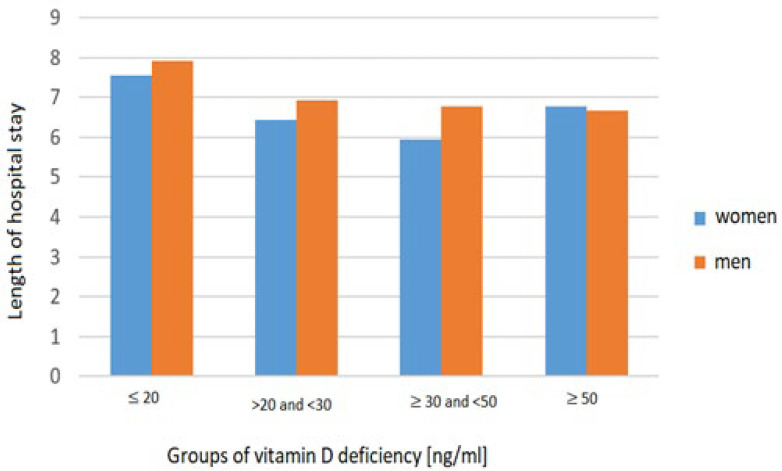
Impact of vitamin D deficiency on LOS in women and men.

Patients who were hospitalized for less than 7 days had an average vitamin D concentration of 24.18 ng/mL, while patients who were hospitalized for more than 7 days had an average concentration of 19.93 ng/mL. In the vitamin D-deficient group, the average LOS was the longest (Figure 4).

**Figure 5:**
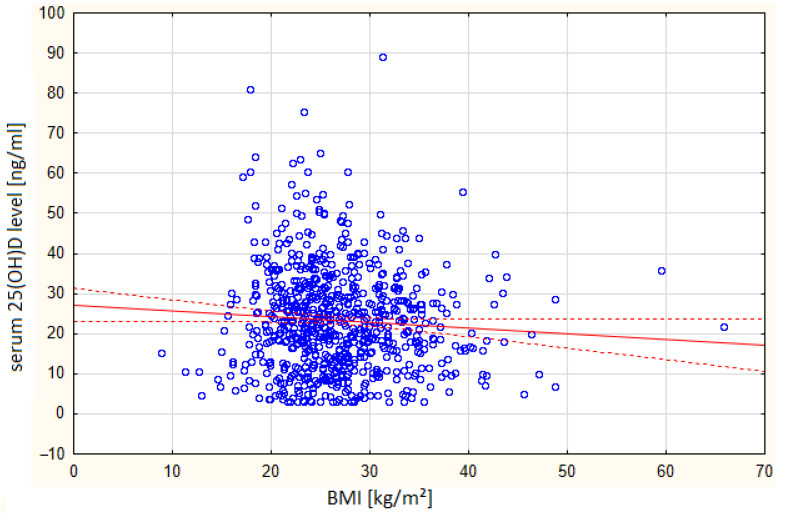
Correlation between BMI and serum vitamin 25(OH)D levels. Confidence interval—95% r = 0.0683.

Patients with severe vitamin D deficiency (≤20 ng/mL) had the longest hospital stays (7.74 days), whereas those with adequate or optimal levels had shorter stays (6.23–6.73 days). This difference was statistically significant (*p* < 0.01). The correlation between BMI and serum vitamin 25(OH)D levels is presented in Figure 5.

In this study, a statistically significant correlation was observed between BMI and vitamin D levels. Patients with vitamin D deficiency had a higher mean BMI (27.17 kg/m^2^) compared to other groups, with the lowest BMI recorded in the group with high vitamin D (23.7 kg/m^2^). The difference was statistically significant in women (*p* = 0.03), but not in men (*p* = 0.08).

We analyzed the correlations between the selected laboratory parameters measured at admission and serum vitamin D concentrations (Table 1). Statistically significant associations were observed for several parameters, including folic acid, total protein, creatinine, aspartate aminotransferase, albumin, red blood cell (RBC) count, high-density lipoprotein (HDL) cholesterol, and total calcium, all of which showed a positive correlation with vitamin D levels. In contrast, creatine kinase-MB (CK-MB), triglycerides, low-density lipoprotein (LDL), cholesterol, and total cholesterol exhibited a negative correlation, with higher values associated with lower vitamin D levels (negative correlation). No statistically significant correlations were found between vitamin D levels and parameters such as glycated hemoglobin (HbA1c), ionized calcium, mean corpuscular volume (MCV), vitamin B12, iron, potassium (K+), and sodium (Na+). The relationship between vitamin D levels, gender, and the presence of common diseases associated with vitamin D deficiency, such as T2DM, arterial hypertension (AH), and osteoporosis, are presented in Table 2.

**Figure 6:**
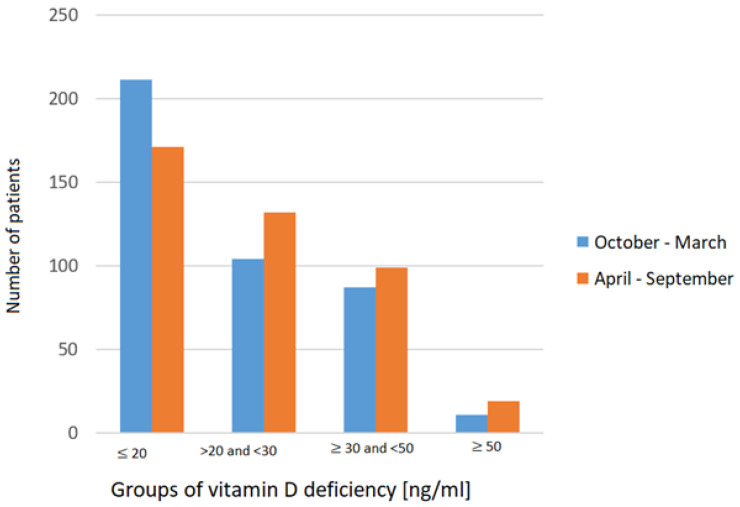
Comparison of vitamin D levels across seasons: October–March vs. April–September.

Severe vitamin D deficiency (≤20 ng/mL) was observed in 53% of men and 40% of women, a difference that was statistically significant (*p* < 0.01). Among women, the distribution across all vitamin D categories was more balanced, whereas men were more likely to exhibit severe deficiency. Additionally, vitamin D deficiency was present in 53% of patients with T2DM, 46% of those with AH, and 31% of those with osteoporosis. However, these associations were not statistically significant (*p* = 0.42, *p* = 0.31, and *p* = 0.17, respectively). This study also examined whether the season of hospitalization influenced serum vitamin D levels. Patients were divided into 2 groups based on the season of admission: October to March (fall and winter months) and April to September (spring and summer months). We found that 211 individuals were vitamin D deficient (≤ 20 ng/mL) during the winter months compared to 171 during the summer months. Conversely, the number of patients with optimal vitamin D levels (30–50 ng/mL) increased from 87 in winter to 99 in summer. However, seasonal variation did not have a statistically significant effect on vitamin D levels (*p* = 0.24) (Figure 6).

**Figure 7:**
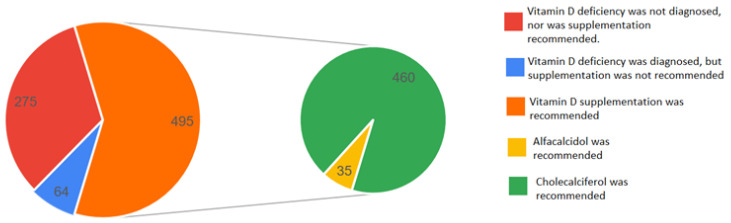
Vitamin D deficiency diagnosis and supplementation recommendations.

The vitamin D levels and the associated medical measures in the patients are presented in Table 3 and Figure 7.

In total, 71% of patients with vitamin D deficiency (≤20 ng/mL) were correctly diagnosed. However, only 26% of these individuals were taking a vitamin D supplement before hospitalization, highlighting a gap in preventive care. In comparison, 32% of patients with moderate deficiency (30–50 ng/mL) reported prior supplementation. At discharge, 61% of patients with vitamin D deficiency received adequate supplementation. The most frequently prescribed form was cholecalciferol (60%), followed by alfacalcidol.

## 4. Discussion

### 4.1. Vitamin D Deficiency

Vitamin D deficiency is a frequently underdiagnosed condition that has gained worldwide attention [10], with an estimated prevalence of 50–90% [3]. Over billions of people worldwide have deficient or inadequate vitamin D levels [2]. In this study, we found that vitamin D deficiency was a significant problem in our patients. The majority (74%) of them had inadequate vitamin D levels. Only 26% of the patients had optimal or high vitamin D levels. A similar study by Karki A et al. [2] claims that vitamin D deficiency (vitamin D levels below 20 ng/mL) was found in 59.39% of patients presenting to the outpatient Department of Medicine of a tertiary care center. In the study conducted by Khattri JB et al., the prevalence of low vitamin levels was found to be 81.8% (vitamin D levels below 30 ng/mL) [10]. In this study, women had higher vitamin D concentrations (mean: 24.4 ng/mL) than men (mean: 20.72 ng/mL). However, Karki A et al. claim that vitamin D deficiency is more common in women (52.09%) [2]. Al-Othman A et al. [9] claim that of the total number of female patients, 52.61% were deficient and only 21.08% of male patients were vitamin D deficient. In a study conducted at the Manipal College of Medical Sciences, the average vitamin D level was low in the age group above 60 years compared to the other age groups. The prevalence of low vitamin D levels was higher in women (90.3%) than in men (68.0%) [10]. A cross-sectional study conducted among 2158 patients in western Nepal found that 73.68% were vitamin D deficient, while only 26.32% had optimal vitamin D levels. Women were 5.29 times more likely to be deficient than men [11].

### 4.2. Length of Hospital Stay (LOS)

The increasing proportion of older people among hospitalized patients poses significant challenges to healthcare systems. This demographic change contributes to increased LOS, which is associated with a range of adverse outcomes, including hospital-acquired infections, functional decline, and increased healthcare costs [14]. Identifying modifiable factors that contribute to prolonged LOS is therefore essential for improving both patient outcomes and the efficiency of hospital care delivery.

Vitamin D deficiency has emerged as one such factor, particularly in older adults, where it has been associated with greater morbidity, impaired physical function, and increased risk of complications [1].

In our study, a statistically significant inverse correlation was observed between serum 25(OH)D levels and LOS, suggesting that a lower vitamin D status may contribute to longer hospitalization.

This finding is consistent with previous studies. Nowak et al. [1] reported that lower serum 25(OH)D concentrations in hospitalized geriatric patients were significantly associated with prolonged hospital stays, proposing vitamin D status as a potential predictor of LOS. Similarly, Hélard et al. [3] identified a linear inverse relationship between serum 25(OH)D and LOS in a geriatric acute care unit, where patients with levels above 50 nmol/L were hospitalized an average of three days less than those with lower levels.

In critically ill patient populations, pre-hospital vitamin D deficiency may predict both short-term and long-term all-cause mortality [13,15]. Blay B et al. further emphasized the association between vitamin D deficiency, increased complication rates, and longer hospital stays, particularly among patients with burns [6]. Beauchet et al. pointed out that the risk of prolonged LOS is highest when vitamin D deficiency coexists with male sex [14], while McWilliams et al. [14,16] reported that nursing home residents with low vitamin D levels were more likely to experience extended hospitalization. Additionally, Boccardi et al. [4] linked hypovitaminosis D correlates with elevated inflammatory markers, which may exacerbate comorbidities and contribute to longer hospitalizations. In orthopedic settings, Maier GS et al. [8] demonstrated a significant inverse association between vitamin D levels and LOS, with patients maintaining adequate vitamin D levels spending 4.3 fewer days in hospital compared to those with deficiency.

Taken together, these findings support the role of vitamin D status as a clinically relevant and modifiable factor influencing hospital length of stay. Routine assessment and management of vitamin D deficiency in hospitalized older adults may therefore be a valuable strategy to enhance recovery, reduce complications, and optimize the use of healthcare resources.

### 4.3. Vitamin D Deficiency and BMI

The published literature has consistently demonstrated an inverse relationship between BMI and serum 25(OH)D concentrations [6]. In the present study, a statistically significant correlation was found between BMI and vitamin D levels.

These findings are consistent with those of previous studies. Al-Othman et al. [9] identified elevated BMI (particularly >30 kg/m^2^) as one of the most significant modifiable predictors of vitamin D deficiency. Several mechanisms have been proposed to explain this relationship, including volumetric dilution, sequestration of vitamin D in adipose tissue, and altered metabolism. These results underline the importance of considering BMI as a key factor when assessing vitamin D status and designing targeted interventions for at-risk populations. The correlation of other laboratory parameters measured upon admission with vitamin D levels was analyzed.

In this study, several biochemical parameters were found to be significantly associated with serum vitamin D levels. Specifically, folic acid, total protein, creatinine, aspartate aminotransferase (AST), albumin, red blood cell (RBC) count, high-density lipoprotein (HDL) cholesterol, and total calcium demonstrated statistically significant positive correlations with vitamin D levels. Conversely, increased levels of CK-MB, triglycerides, LDL cholesterol, and total cholesterol were negatively correlated with vitamin D status. No statistically significant correlation was found between vitamin D levels and other laboratory parameters, including HbA1c, ionized calcium, MCV, vitamin B12, iron, K+, and Na+. These findings are consistent with previously published data that highlight the complex metabolic role of vitamin D and its association with markers of nutritional and cardiovascular health [5,16].

Vitamin D exerts pleiotropic effects on various metabolic pathways, including lipid metabolism, calcium homeostasis, and protein synthesis. Low vitamin D concentration is often associated with increased inflammation and oxidative stress, which may contribute to unfavorable lipid profiles and poorer overall metabolic health [5]. Individuals with sufficient vitamin D levels tend to exhibit lower concentrations of triglycerides, LDL cholesterol, and total cholesterol. In contrast, vitamin D-deficient individuals tend to present higher adiposity, a factor strongly associated with dyslipidemia [5]. Mc Williams C et al. [16] reported that approximately 90% of individuals with serum vitamin D concentrations below 30 ng/mL were receiving pharmacologic treatment for dyslipidemia, further supporting a potential association between vitamin D deficiency and adverse lipid profiles.

In terms of calcium metabolism, vitamin D plays a critical role in facilitating intestinal calcium absorption. A deficiency in vitamin D may result in secondary hyperparathyroidism, bone loss, and an increased risk of fractures, particularly in older adults [5,6,11]. Our findings showed a positive correlation between total calcium levels and serum vitamin D concentrations, consistent with the physiological role of vitamin D in promoting calcium absorption. What is more, a significant positive correlation between albumin and vitamin D was observed, which mirrors findings in other studies. For instance, Blay et al. [6] reported that patients with vitamin D deficiency had significantly lower levels of total and ionized calcium, as well as albumin. Furthermore, Abel et al. [4] demonstrated an association between hypoalbuminemia and low serum vitamin D levels, suggesting that the coexistence of these abnormalities may help to identify a high-risk group of geriatric patients who require more intensive monitoring and intervention. Interestingly, despite some earlier data, they found no significant correlation between vitamin D and sodium Na + or creatinine. However, positive correlations were observed for folic acid, albumin, and total calcium, which is consistent with our results. A lack of correlation between vitamin D levels and Na+ was consistently reported in both our study and prior research.

In summary, these results emphasize the relevance of vitamin D as a biomarker of nutritional and metabolic health and suggest that several commonly measured biochemical parameters may reflect or be influenced by serum vitamin D concentrations. Further research is needed to clarify the cause–effect relationships and mechanisms underlying them.

### 4.4. Comorbidities

Regarding comorbidities, although vitamin D deficiency has been implicated in the development of chronic diseases such as T2DM, AH, and osteoporosis [2,5,7], our study did not find statistically significant associations between vitamin D levels and the presence of these conditions (Table 2). Similar results were reported by McWilliams C et al. [16], who noted that despite a high prevalence of vitamin D deficiency in individuals with T2DM, AH, and osteoporosis, no significant statistical correlations were established. Nonetheless, several studies have failed to demonstrate a consistent antihypertensive effect of vitamin D, thereby questioning its role in blood pressure regulation [7]. Nevertheless, other studies suggest that vitamin D supplementation may confer metabolic benefits. For instance, vitamin D has been shown to reduce the risk of progression from prediabetes to diabetes by 15% and increase the likelihood of regression to normoglycemia by 30%, with no observed adverse effects [5].

In our study population, among individuals with vitamin D deficiency, 53% had T2DM and 46% had AH. These findings are consistent with data presented by Karki et al. [2], who reported T2DM in 25.6% and hypertension in 53.5% of vitamin D-deficient patients.

In summary, while our study did not find statistically significant associations between vitamin D levels and chronic conditions such as T2DM, AH, and osteoporosis, the high prevalence of these comorbidities among vitamin D-deficient individuals aligns with previous research, highlighting the potential but inconclusive link between vitamin D deficiency and metabolic disorders.

### 4.5. Vitamin D Levels and the Month of Hospitalization

This study aimed to determine whether the time of the year during which hospitalization occurred had a significant impact on serum vitamin D levels. The research was conducted in Silesia, a region located in southern Poland (latitude 50°N), where cutaneous synthesis of vitamin D occurs predominantly from late April through early September. During the months from October to March, UVB radiation levels are insufficient to support effective endogenous vitamin D synthesis. Seasonal fluctuations in vitamin D status—particularly deficiencies during winter—have been widely reported in the literature [17]. However, our findings did not reveal a statistically significant variation in vitamin D levels among the study population (*p* = 0.24). Similar results were presented by Nowak et al. [17], who also reported a lack of seasonal fluctuation in specific patient populations. A plausible explanation for this finding is the age-related decline in vitamin D synthesis. With age, the skin synthesis of 7-dehydrocholesterol decreases, which in turn reduces the capacity for vitamin D synthesis—by up to 75% in individuals aged 70 compared to those aged 20. Consequently, older adults often exhibit persistently low vitamin D levels, irrespective of seasonal changes. In contrast, other studies have reported significant seasonal variations. For instance, Bhatta MP et al. [11] found that vitamin D deficiency increases in winter (81.62%) and spring (88.82%) compared to fall (57.07%) and summer (70.39%) (*p* = 0.00). The discrepancy between these findings and ours may be attributed to differences in the study populations, particularly regarding age, comorbidities, and lifestyle factors such as sun exposure and dietary intake.

In summary, although seasonal variation in vitamin D status is well documented in the general population, our study did not identify a significant association between the season of hospitalization and serum vitamin D levels, most likely due to the advanced age of the study cohort and the resulting impaired cutaneous synthesis of vitamin D.

### 4.6. Vitamin D Supplementation

It is estimated that up to 90% of the Polish population suffers from vitamin D deficiency, which highlights the importance of following current guidelines on appropriate supplementation [13]. Daily vitamin D regimens are the most efficient and beneficial strategy for improving vitamin D status [5], given their safety, simplicity, and potential to reduce LOS [8].

In the present study, we assessed serum vitamin D levels and corresponding medical interventions in patients (Table 3). According to current clinical guidelines, serum vitamin D concentrations of 20 ng/mL or below indicate a deficiency requiring immediate medical treatment with therapeutic dosing, while concentrations greater than 20 ng/mL but less than 30 ng/mL reflect a suboptimal status warranting a moderate increase in dosing. Levels between ≥30 ng/mL and 50 ng/mL are considered adequate to optimal, while concentrations greater than 50 ng/mL and up to 100 ng/mL indicate a high vitamin D supply [12]. Among our study population, 71% of patients were classified as deficient and 28% as suboptimal. Despite the high prevalence of deficiency, only 61% of those in the deficient group received a documented recommendation for vitamin D supplementation, suggesting that 39% of them did not receive appropriate intervention. Similar results were presented by Blay B et al. [6], who observed that 42.7% of patients with low vitamin D levels did not receive supplementation during hospitalization.

Although vitamin D is widely available over the counter, as both dietary supplements and pharmaceutical preparations, only 118 (14%) patients reported taking vitamin D supplements prior to hospitalization. In this study, physicians most recommended supplementation with cholecalciferol (N = 460), followed by alfacalcidol (N = 35). Cholecalciferol remains the preferred form of vitamin D supplementation, given its favorable safety profile and effectiveness in maintaining adequate serum 25(OH)D levels. In contrast, active analogs such as calcifediol, calcitriol, alfacalcidol, as well as parenteral forms are typically reserved for patients with impaired vitamin D metabolism, such as those with renal failure or hypoparathyroidism, due to the increased risk of hypercalcemia and hypercalciuria [10,13].

A daily or cumulative dosing regimen (weekly, biweekly, or monthly) of cholecalciferol is safe and effective. The recommended dose for healthy adults aged 19–65 years is 1000–2000 IU/day (25–50 µg/day) with similar dosages advised for individuals aged 66–75 years. For those aged over 76 years, the recommended intake increases to 2000–4000 IU/day (50–100 µg/day). Overweight and obese individuals require double the standard recommended dose for their age group [12].

Alfacalcidol, a vitamin D active analogue frequently used to treat postmenopausal and glucocorticosteroid-induced osteoporosis (GIO), is also indicated for men with osteoporosis. The recommended dose is 0.5 to 1 µg/day. In Poland, alfacalcidol is approved for the treatment of osteoporosis with coexisting vitamin D deficiency, hypocalcemia, vitamin D-resistant rickets, osteomalacia, hypoparathyroidism, and calcium–phosphorus imbalance in chronic kidney disease. Due to its pharmacological activity, monitoring of serum calcium is essential both prior to initiation and 3–6 months after treatment onset [13].

To sum up, despite the high prevalence of vitamin D deficiency among patients, appropriate supplementation is often underutilized, underscoring the need for improved adherence to clinical guidelines and more proactive intervention strategies to ensure effective prevention and management of deficiency-related health risks.

Limitations: This study had several limitations.

Medication data were collected at enrollment through patient interviews, which may lead to inaccuracies. Some patients were unable to report the exact form or dosage of vitamin D they were taking; therefore, this information was not included in the analysis. Recommendations for supplement use were made by physicians at discharge, allowing for a distinction between supplements and pharmaceuticals; however, this may not fully reflect the actual reasons for prescribing decisions.

This was a retrospective study based on hospitalized patients, which limited the ability to establish causal relationships. Of the 996 patients admitted in 2022, 162 were excluded due to missing data on vitamin D status. This exclusion may have led to selection bias. In addition, the exact reasons for measuring vitamin D levels in the included patients could not be determined, as these measurements were often part of a broader diagnostic process for various conditions.

The study did not collect information on the reasons for prescribing or not prescribing vitamin D at discharge. Only the presence or absence of a prescription was recorded. It can therefore only be surmised that the lack of prescription could be related to the lack of awareness of this issue among doctors or their lack of attention to it.

The lack of data on the primary cause of hospitalization and the severity of comorbidities limits the ability to fully interpret the observed associations. Additionally, the relatively small sample size may reduce the generalizability of the findings. Further studies should include assessment of frailty, including standardized assessment of mobility and functional status. Establishing a direct causal relationship between vitamin D levels and length of hospitalization is inherently challenging due to the influence of multiple potential confounding variables. These may include the patient’s functional status, severity of illness, cognitive function, nutritional status, comorbidity burden, specific diagnoses, polypharmacy, age, and sex.

Taking these factors into account in future studies will be essential to better understand the complex relationship between vitamin D status and clinical outcomes in hospitalized patients.

## 5. Conclusions

Vitamin D deficiency remains a common condition with serious health consequences, especially among hospitalized patients. Despite the widespread availability of effective treatment, gaps in clinical practice remain—39% of people with confirmed vitamin D deficiency do not receive recommended supplementation. This deficiency is associated with prolonged hospitalization. Vitamin D may potentially have a role as a cost-effective and accessible marker for identifying hospitalized patients at increased risk for poor clinical outcomes.

Low vitamin D levels were associated with unfavorable lipid profiles and were consistently observed in patients with higher BMI, indicating the need for individual supplementation strategies. Moreover, women generally had higher average vitamin D levels than men, and there was no significant seasonal variation, especially among older adults. Although a significant proportion of patients with a deficiency also presented with hypertension (46%) and T2DM (53%), no direct statistical association with vitamin D levels was established. Only 14% of patients reported prior use of vitamin D supplements, underscoring the need for increased public awareness and preventative measures. Cholecalciferol was the preferred form of supplementation. However, many patients still had values that were in the deficient or insufficient range, suggesting that current dosing strategies may be insufficient and require optimization. Routine vitamin D screening and appropriate supplementation should be a priority, especially in older adults, those with higher BMI, and those with chronic diseases. Public education on the consequences of vitamin D deficiency and its integration into standard healthcare practices is essential. Maintaining adequate vitamin D levels can contribute to shorter hospitalizations, improved metabolic health, and increased functional independence in vulnerable populations.

## Figures and Tables

**Table 1:** Baseline clinical and laboratory characteristics of the study group; N—number of examined patients.

Group Parameter [Units]	Total		Vitamin D ≤ 20 ng/mL		Vitamin D >20 and <30 ng/mL		Vitamin D ≥ 30 and <50 ng/mL		Vitamin D ≥ 50 ng/mL		*p*-Value
	N	Mean Value	N	Mean Value	N	Mean Value	N	Mean Value	N	Mean Value	
Age [years]	834	63.03	382	63.48	236	62.24	186	62.84	30	64.56	0.78
LOS [days]	834	7.06	382	7.74	236	6.64	186	6.23	30	6.73	<0.01
Body weight [kg]	733	75.74	325	77.60	211	75.19	173	73.87	24	68.97	0.90
Height [cm]	734	167.68	325	168.63	212	166.90	172	166.91	25	167.44	0.30
BMI index [kg/ m^2^]	726	26.80	321	27.17	210	26.95	171	26.36	24	23.70	0.05
HbA1c [%]	213	7.06	116	7.36	51	6.70	40	6.77	6	6.06	0.12
Albumin [g/dL]	243	3.55	122	3.21	65	4.11	48	3.72	8	3.22	<0.01
Total protein [g/dL]	728	6.37	333	6.07	203	6.56	169	6.68	23	6.80	<0.01
Ionized calcium [mmol/L]	236	1.34	107	1.37	69	1.38	52	1.26	8	1.27	0.37
Total calcium [mg/dL]	747	9.20	343	9.05	208	9.36	170	9.31	26	9.36	<0.01
Alanine aminotransferase (ALT) [IU/L]	797	34.70	361	38.28	231	30.28	178	33.01	27	35.70	0.06
Aspartate aminotransferase (AST) [IU/L]	796	35.82	361	40.72	230	31.83	178	31.32	27	34.11	<0.01
Creatinine [mg/dL]	814	1.03	373	1.04	227	0.99	184	1.05	30	1.03	<0.01
Natrium (Na+) [mmol/L]	834	138.44	382	138.01	236	138.26	186	138.41	30	138.56	0.54
Potassium (K+) [mmol/L]	832	4.35	380	4.34	236	4.38	186	4.34	30	4.34	0.16
Hemoglobin [g/dL]	824	12.24	378	11.79	233	12.50	183	12.88	30	12.11	<0.01
Red blood cells (RBCs) [mln/μL]	823	4.29	378	4.18	233	4.57	182	4.19	30	4.07	<0.01
Iron [ug/dL]	217	66.54	106	66.56	61	74.71	43	58.23	7	46.07	0.43
Mean corpuscular volume (MCV) [fl]	824	90.70	378	91.38	233	89.90	183	90.68	30	88.50	0.27
Vitamin B12 [pg/mL]	196	510.88	101	492.99	51	494.33	41	576.58	3	497.00	0.93
Folic acid [ng/mL]	135	10.35	69	8.08	38	14.82	27	10.21	1	5.89	<0.01
Total cholesterol [mg/dL]	805	159.87	366	150.47	230	166.46	179	170.83	30	158.67	<0.01
Triglycerides [mg/dL]	791	121.81	358	129.06	228	119.06	176	112.27	29	111.71	0.05
HDL cholesterol [mg/dL]	709	48.40	319	42.79	204	51.60	160	55.47	26	48.47	<0.01
LDL cholesterol [mg/dL]	697	90.89	309	84.09	202	93.04	159	100.90	27	93.35	0.03
Creatine kinase-MB (CK-MB) [IU/L]	422	129.31	198	147.88	117	124.14	92	106.47	15	64.40	0.02

**Table 2:** The relationship between vitamin D levels and the presence of common diseases associated with vitamin D deficiency (DM2, AH, osteoporosis); N—number of examined patients, F—frequency of a given disease in a given group.

Group Disease	Total	Vitamin D ≤ 20 ng/mL		Vitamin D > 20 and <30 ng/mL		Vitamin D ≥ 30 and <50 ng/mL		Vitamin D ≥ 50 ng/mL		*p*-Value
	N	N	F [%]	N	F [%]	N	F [%]	N	F [%]	
T2DM	255	134	53	68	27	44	17	9	4	0.42
AH	512	234	46	137	27	124	24	17	3	0.31
Osteoporosis	42	13	31	16	38	10	24	3	7	0.17

**Table 3:** The relationship between vitamin D levels and diagnosis of vitamin D deficiency by physicians, use of vitamin D supplementation before hospitalization, recommendations for vitamin D supplementation at discharge by physicians, and the use of two specific forms of vitamin D (cholecalciferol and alfacalcidol); N—number of examined patients, F—frequency of a given management in a given group.

Group Management	Total	Vitamin D ≤ 20 ng/mL		Vitamin D > 20 and <30 ng/mL		Vitamin D ≥ 30 and <50 ng/mL		Vitamin D ≥ 50 ng/mL	
	N	N	F [%]	N	F [%]	N	F [%]	N	F [%]
Diagnosis of vitamin D deficiency by physicians	415	293	71	115	28	6	1	1	0
Taking vitamin D before hospitalization	118	31	26	37	31	38	32	12	10
Recommendation of vitamin D at discharge	495	302	61	140	28	45	9	8	2
Recommendation of cholecalciferol	460	276	60	133	29	43	9	8	2
Recommendation of alfacalcidol	35	26	74	7	20	2	6	0	0

## Data Availability

The original contributions presented in the study are included in the article, further inquiries can be directed to the corresponding authors.

## Abbreviations

The following abbreviations are used in this manuscript:

LOS	length of hospital stay
25(OH)D	25-hydroxyvitamin D
T2DM	type 2 diabetes
AH	arterial hypertension
